# “Reading” a new chapter in protozoan parasite transcriptional regulation

**DOI:** 10.1371/journal.ppat.1010056

**Published:** 2021-12-02

**Authors:** Krista Fleck, Malorie Nitz, Victoria Jeffers

**Affiliations:** Molecular, Cellular and Biomedical Sciences, University of New Hampshire, Durham, New Hampshire, United States of America; Joan and Sanford I Weill Medical College of Cornell University, UNITED STATES

## Abstract

Protozoan parasites continue to cause a significant health and economic burden worldwide. As infectious organisms, they pose unique and difficult challenges due to a level of conservation of critical eukaryotic cellular pathways with their hosts. Gene regulation has been pinpointed as an essential pathway with enough divergence to warrant investigation into therapeutically targeting. Examination of human parasites such as *Plasmodium falciparum*, *Toxoplasma gondii*, and kinetoplastids have revealed that epigenetic mechanisms play a key role in their gene regulation. The enzymes involved in adding and removing epigenetic posttranslational modifications (PTMs) have historically been the focus of study. However, the reader proteins that recognize and bind PTMs, initiating recruitment of chromatin-modifying and transcription complexes, are now being realized for their critical role in regulation and their potential as drug targets. In this review, we highlight the current knowledge on epigenetic reader proteins in model parasitic protozoa, focusing on the histone acyl- and methyl-reading domains. With this knowledge base, we compare differences between medically relevant parasites, discuss conceivable functions of these understudied proteins, indicate gaps in knowledge, and provide current progress in drug development.

## Introduction

Infectious disease remains a significant public health challenge worldwide, causing devastating morbidity and mortality, a burden carried predominantly by developing countries. Parasitic infections caused by protozoan organisms signify a particularly difficult challenge, and despite some eradication efforts, parasitic infections remain endemic in many regions. Rapid emergence of multidrug-resistant parasites and the lack of suitable drug targets continues to hinder control measures.

Regulation of gene expression is an essential and therapeutically targetable process that is generally well conserved among eukaryotic organisms. Epigenetic machinery plays the critical roles of marking genes for transcription or repression and recruiting chromatin-modifying and transcription complexes. The basic unit of chromatin is the nucleosome, which consists of 147 base pairs of DNA wrapped around an octamer of the 4 core histones H2A, H2B, H3, and H4. To control accessibility of DNA for gene expression, chromatin structure is modulated by different epigenetic mechanisms. One way in which the chromatin structure may be altered is through posttranslational modifications (PTMs) of the histones, which can include acylation, methylation, phosphorylation, and ubiquitination [1,2]. These PTMs can recruit machinery to initiate nucleosome repositioning or swapping of core and variant histones, thus facilitating a structure permissive or prohibitive to active gene expression. The primary mediators of signaling between histone modifications and transcriptional regulatory complexes are the PTM reader proteins. Thus, they fulfill a critical function in epigenetic regulation of transcription. The 3 major histone modifications covered in this review include acetylation, methylation, and phosphorylation.

The acetylation of lysine residues on histones is a reversible process and is regulated by histone acetyltransferases (KATs) and histone deacetylases (HDACs). KATs catalyze the addition of an acetyl group from acetyl-CoA to the ε-amino group of a lysine residue, neutralizing the positive charge on the lysine. HDACs reverse this reaction and restore the positive charge on lysine residues [3]. The neutralization of the positive lysine by acetylation weakens the interaction with negatively charged DNA, resulting in a looser, less compacted chromatin structure, making the DNA accessible to regulatory complexes.

Histone methylation can occur on lysine and arginine residues. Lysine residues may be mono-, di-, or tri-methylated, and arginine residues may be mono- or di-methylated. Several histone methyltransferases and demethylases have been identified that catalyze the addition and removal of a methyl group on free amino groups of target residues. In contrast to histone acetylation, which is generally associated with activation of gene expression, histone methylation may activate or repress transcription in a context-dependent manner [4].

Histone phosphorylation may occur on serine, threonine, or tyrosine residues but is generally less abundant than the other major histone modifications [1]. Phosphorylation of histones increases the negative charge of the nucleosome, resulting in a more relaxed chromatin state [5]. Phosphorylation is mediated by many kinases and phosphatases, which phosphorylate and dephosphorylate residues, respectively. Histone phosphorylation plays a major role in DNA damage repair; however, many studies have also cited its role in transcriptional regulation and chromatin compacting [1,6].

Many of the organisms responsible for parasitic infections in humans possess sophisticated epigenetic mechanisms, which aid in regulating their gene expression for establishing virulence, persisting in stressful conditions, and adapting to different hosts and transmission routes. Fortunately, these regulatory mechanisms are sufficiently divergent from humans to warrant closer inspection with the goal of identifying unique features that may support the development of effective, nontoxic therapies for treating parasitic infections. While the enzymatic regulators (writers and erasers) have historically been the focus of investigation, the adaptor proteins that bind PTMs on histones (readers) have more recently been identified as critical components of gene regulation that have therapeutic potential for protozoan infections.

Here, we review the known epigenetic reader domains and identify the proteins that contain these domains in a number of major protozoan pathogens. We also discuss the ongoing efforts to understand the function of reader proteins in protozoan species of significance such as the malaria parasite *Plasmodium falciparum*, *Toxoplasma gondii*, and the kinetoplastid parasites, *Trypanosoma brucei* and *Trypanosoma cruzi*. Importantly, we highlight the gaps in what we know about these proteins in additional organisms of significance to human and animal health, such as *Cryptosporidium* sp., *Leishmania* sp., *Entamoeba histolytica*, *Theileria annulata*, *Giardia* sp., and *Trichomonas vaginalis*.

### Lysine acylation

Lysine acylation has emerged as a key posttranslational regulatory mechanism in eukaryotic organisms. Of the possible acyl modifications, lysine acetylation is by far the most commonly observed and studied [3]. This neutralizes the positive charge on the lysine residue, which can alter protein structure and protein–protein or protein–DNA interactions [7]. Although lysine acetylation occurs predominantly on histones, proteome-wide analysis in many different species including protozoans reveals that it also occurs on non-histone proteins, implicating it in a variety of cellular processes, such as metabolism, RNA processing, protein translation, cellular structure, and transport (reviewed in [8]). Domains that can recognize and “read” lysine acyl marks include the bromodomain, YEATS domain, and double PHD finger (DPF) domains. Protozoan parasites possess conserved bromodomains and YEATS domains; however, DPF domains have yet to be identified (Table 1).

**Table 1:** Reader domains of histone PTMs in protozoan parasites. Green shading indicates the presence of a particular reader domain in the corresponding species.

Histone PTM	Reader domain	*Plasmodium falciparum*	*Toxoplasma gondii*	*Cryptosporidium parvum*	*Theileria annulata*	*Trypanosoma brucei*	*Trypanosoma cruzi*	*Leishmania major*	*Giardia lamblia*	*Entamoeba histolytica*	*Trichomonas vaginalis*	Citations
Lysine acylation	Bromodomain											[11–25,33,35,40–42]
	DPF											
	YEATS											[33,53]
Lysine methylation	ADD											
	Ankyrin											
	Chromobarrel											
	Chromodomain											[33,80,83,85,112]
	Double chromodomain											
	PHD											[33,83,94,95]
	PWWP											[33,99]
	Tudor											[89]
	WD40											
	Zinc finger CW											[33]
Arginine methylation	ADD											
	Tudor											[89]
	WD40											
Phosphorylation	14-3-3											[103,113]
	BIR											
	BRCT											

DPF, double PHD finger; PHD, plant homeodomain; PTM, posttranslational modification.

### Bromodomains

**Fig 1:**
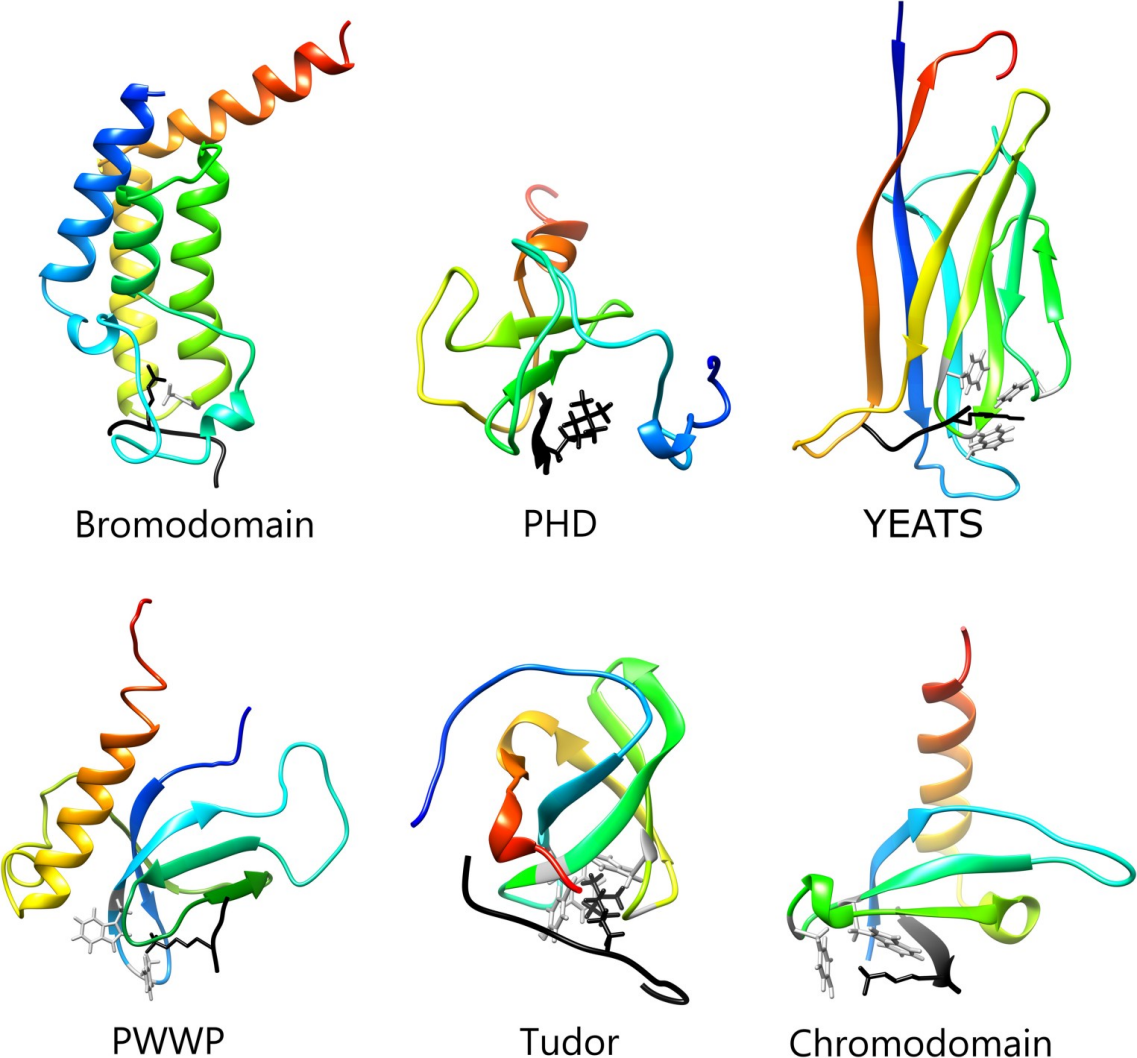
Structures of reader domains from protozoan parasites. Experimentally derived or computationally predicted structures of 6 reader domains. The bound histone peptide is depicted in the binding pocket of each domain as a black ribbon with the target PTM. Residues in the reader domain that coordinate binding of the target PTM are shown in gray, noting that PHDs do not have specific residues required for binding. In clockwise order: Bromodomain, *Plasmodium falciparum* PfBDP1 (PDB: 7M97); PHD, *P*. *falciparum* PfSET1, predicted structure; YEATS, *Toxoplasma gondii* TgGAS41, predicted structure; Chromodomain, *P*. *falciparum* PfHP1, predicted structure; Tudor *Cryptosporidium parvum* cgd4_270, predicted structure; PWWP *Trypanosoma brucei* TbTFIIS-2 (PBD:2NAS). Predictions made using I-TASSER unbiased threading [114]. PHD, plant homeodomain; PTM, posttranslational modification.

The bromodomain is the most abundant reader of lysine acetylation, is ubiquitous in eukaryotic organisms, and is the most studied of all reader proteins. Approximately 110 amino acid residues in length, the bromodomain consists of 4 α-helices linked by ZA and BC loop segments [9]. The domain folds into a left-handed bundle forming a hydrophobic pocket for the acetylated lysine residue (Fig 1). The BC loop forms a hydrogen bond with the oxygen of the acetyl-carbonyl group on the acetylated lysine; 4 water molecules link the ZA loop and the acetylated lysine. A highly conserved asparagine residue within the binding pocket is required for acetyl-lysine recognition [9,10]. The ZA and BC loops are of variable sequence and length, providing specificity for the target acetyl-lysine residue [10]. Bromodomains have been identified as critical regulators of gene expression in many protozoan parasites [11].

**Fig 2:**
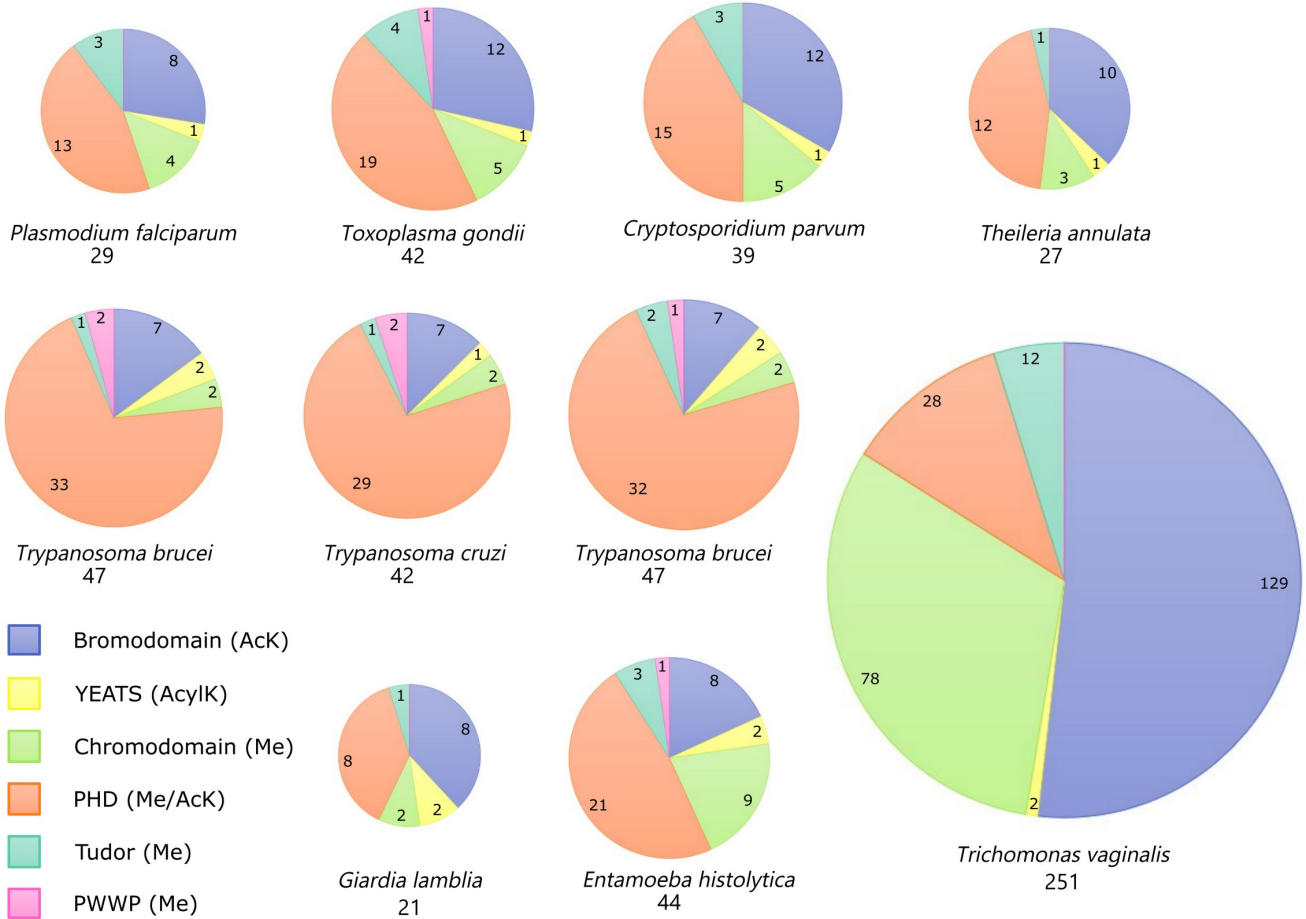
Reader domains in protozoan parasites. Reader domains are found in protozoans across many different phyla. In most organisms, the majority of readers that they encode are PHD proteins (orange), reflecting the versatility of this domain’s function and binding targets. Unusually, *Trichomonas vaginalis* encodes a large expansion of reader domain proteins, most notably of bromodomains and chromodomains. The reader domain containing proteins were identified by Interpro domain homology searches on VEuPathDB (https://veupathdb.org/) from 10 parasite species. The size of each pie chart is scaled relative to the total number of reader domain proteins in each species. Numbers in pie charts indicate total number of proteins containing each domain and numbers below chart represent total number of all reader proteins in each parasite. AcK, acetylated lysine; AcylK, acylated lysine; Me, methylation; PHD, plant homeodomain.

The *P*. *falciparum* genome encodes 8 bromodomain-containing proteins (Fig 2 and S1 Table), of which 4 are homologous to proteins found in humans and yeast [11,12]. This includes PfGCN5, a member of the GNAT family of lysine acetyltransferases, with a conserved domain architecture consisting of a KAT domain and a C-terminal bromodomain. PfGCN5 has been shown to preferentially acetylate lysine residues on histone H3 through the enzymatic function of the KAT domain [13,14]. The role of the bromodomain in PfGCN5 function is currently unknown, but it is clear from mutagenesis experiments that it plays an important role. Deletion of the bromodomain of PfGCN5 resulted in significant reduction of H4K9ac and H4K14ac levels [15]. This decrease in histone acetylation levels led to the dysregulation of over 60% of parasite genes. Despite the substantial effect of PfGCN5 bromodomain removal on parasite gene expression, parasites were still viable, indicating that while the bromodomain is important for parasite growth, it is not essential for survival. Further study of PfGCN5 bromodomain deletion mutants revealed growth defects that were associated with dysregulation of gene expression and modified chromatin states due to the reduction in histone acetylation, leading to altered cellular pathways [15]. RNA-seq and ChIP-seq analyses found that PfGCN5 is significantly up-regulated under stress conditions and binds to the promoter region of stress-responsive genes, respectively [16]. This suggests that PfGCN5 drives the parasite stress response by placement and recognition of acetylated lysine residues but is not required for parasite survival.

There are 4 bromodomain-containing proteins in the *P*. *falciparum* genome that are unique to Apicomplexan parasites [11]. *P*. *falciparum* BromoDomain Proteins 2, 3, and 4 (BDP2, BDP3, BDP4) remain uncharacterized, and, in the absence of other functional domains, their function is unpredictable. PfBDP1 contains a C-terminal bromodomain and several ankyrin repeats that likely mediate protein–protein interactions [17]. Inducible knockdown of PfBDP1 led to down-regulation of almost all 75 annotated merozoite invasion–related genes, and parasites were duly deficient in erythrocyte invasion [18]. In vitro binding experiments determined that PfBDP1 binds to H3K9ac and H3K14ac [18]. However, ChIP analysis to compare PfBDP1 binding sites with histone acetylation patterns in the parasite chromatin found that although PfBDP1 did colocalize with H3K9Ac enriched at promoters, it was not complete colocalization, indicating that other factors are influencing PfBDP1 recruitment [18]. Indeed, interactome analysis to determine the PfBDP1-associated complex showed that PfBDP1 couples with another bromodomain protein, PfBDP2 that likely also contributes to chromatin binding and recruitment of the complex [18]. Furthermore, the DNA binding protein, PfAP2-I, was identified in the PfBDP1 complex, which was subsequently found to recruit PfBDP1 to invasion-related genes through recognition of a consensus motif found in promoters of a subset of genes significantly enriched in invasion genes [19]. Tang and colleagues further compared ChIP-seq datasets and showed that PfBDP1 and PfAP2-I have similar genomic localization as active gene markers H3K18ac, H3K27ac, and histone variant H2A.Z [20]. Additionally, PfBDP1/PfAP2-I were enriched at the acetylation sites consistent with exposed DNA at active transcriptional start sites of invasion-related genes, suggesting that a PfBDP1-containing complex is likely involved in activation of invasion-related genes in *P*. *falciparum*.

*Toxoplasma* contains 12 bromodomain-containing proteins (Fig 2 and S1 Table), of which 6 are homologous to proteins found in other eukaryotes, while the remaining 6 are unique to parasites in the Apicomplexan phylum [11]. Unusually, *Toxoplasma* expresses 2 distinct homologs of the lysine acetyltransferase GCN5, denoted TgGCN5a and TgGCN5b [21]. Similarly to other GCN5 homologs, both contain a conserved KAT domain and a C-terminal bromodomain, but also have large N-terminal extensions with unknown function, which may be important for protein–protein interactions [21]. TgGCN5a is a functional lysine acetyltransferase that shows strong specificity for acetylation of lysine 18 on histone H3 [21]. Although TgGCN5a is expressed during the tachyzoite stage, it appears to be nonessential as TgGCN5a knockout parasites displayed no phenotype during normal growth conditions [22]. However, parasites lacking TgGCN5a were deficient in responding to alkaline stress and were unable to up-regulate genes required for formation of the dormant bradyzoite stage, indicating an important role for TgGCN5a in parasite development [22]. The ortholog, TgGCN5b, is essential for tachyzoite viability [23]. Expression of a dominant negative TgGCN5b, in which the KAT domain was enzymatically inactive, led to a reduction in histone H3 acetylation, causing global dysregulation of gene expression and an arrest of parasite proliferation [23]. In contrast to *P*. *falciparum*, the bromodomain of TgGCN5b appears to be essential for its function. Expression of an ectopic copy of TgGCN5b in which the bromodomain was mutated to abolish acetyl-lysine binding had a dominant negative effect on parasite growth, suggesting that the bromodomain plays an important role in stabilizing the TgGCN5b complex or recruiting it to the chromatin at target gene loci [24].

The functions of the 6 parasite-specific bromodomain-containing proteins encoded in the *Toxoplasma* genome have yet to be determined. TgBDP1 and TgBDP2 are homologs of PfBDP1 and PfBDP2 and are predicted to be essential for tachyzoite growth [25], but it is unknown if they carry out a similar function to the *P*. *falciparum* homologs. The double bromodomain-containing protein TgBDP3 contains some homology to the single bromodomain in PfBDP3 [11]. Coccidian homologs contain a double bromodomain, while homologs in species in the Aconoidasida class contain only a single bromodomain; this difference indicates a possible divergence in the functions of these proteins in different Apicomplexan species.

Further divergence in bromodomain function between apicomplexan classes can be seen in the expanded repertoire of bromodomain proteins in the Coccidia compared to other Apicomplexan parasites. The *Toxoplasma* proteins TgBDP4 and 5 are conserved in Coccidians but are absent in the Aconoidasida. The *Toxoplasma* bromodomain protein TgBDP6 is a large protein containing several domains that mediate protein–protein or protein–DNA interactions, including WD-40 domains, a plant homeodomain (PHD), and the C-terminal bromodomain. TgBDP6 is similarly conserved within the Coccidia, and related sequences can also be found in the Piroplasms but not the Haemosporidians. Intriguingly, the Piroplasm homologs do not contain a bromodomain in the C-terminal region of the protein, suggesting that the other interaction domains are sufficient for protein function.

The regulatory mechanisms for transcription in trypanosomatids have diverged quite significantly from other eukaryotic organisms. This is evident in the polycistronic organization of genes, divergent histone sequences, and presence of the modified thymidine residue known as BaseJ [26–28]. Nonetheless, these parasites also harness PTM of histone tails to regulate transcription [27–29] and similarly rely on reader proteins to mediate interactions between the chromatin and the transcriptional machinery.

The causative agent of African sleeping sickness, *Tryp*. *brucei*, encodes 7 bromodomain-containing proteins (Fig 2 and S1 Table), TbBDF1-7, which do not display any significant homology to proteins outside of the Kinetoplastida. TbBDF1-6 appear to be involved in initiation of transcription based on gene knockdown and/or genomic localization to transcriptional start sites. In vitro binding experiments determined that TbBDF2 binds the hyperacetylated N-terminus of histone variant H2A.Z [30], consistent with a role in transcriptional activation. TbBDF3 is also recruited to transcriptional start sites, although the histone mark that is responsible for its recruitment to the chromatin is unknown [31–33]. Knockdown of both TbBDF2 and TbBDF3 in *Tryp*. *brucei* blood stages disrupted parasite growth and led to dysregulation of gene expression [32]. Strikingly, knockdown of either TbBDF2 or TbBDF3 resulted in up-regulation of many genes specific to the parasite insect stages and disruption of the mutually exclusive control of the parasite’s repertoire of variant surface glycoproteins (VSGs) [32], underscoring the importance of these bromodomain proteins in establishing and maintaining bloodstream infection. A BDF4 homolog is found in all trypanosomatids sequenced to date and contains 2 N-terminal bromodomains. In *Tryp*. *brucei*, TbBDF4 is currently annotated with only a single bromodomain (https://tritrypdb.org/); however, multiple alignment and protein analysis reveals a second bromodomain sequence that contains the conserved asparagine motif required for acetyl-lysine binding (NCxxY/FN). TbBDF7 contains a bromodomain and an AAA+ ATPase domain and is enriched at transcription termination sites [34]. The protein colocalizes with loci enriched in histones H3.V and H4.V, where RNA polymerase II mediated transcription is terminated. TbBDF7 also interacts with nucleosome assembly proteins TbNAP1 and TbNAP2, suggesting that TbBDF7, TbNAP1, and TbNAP2 cooperate to mediate H3.V/H4.V deposition at transcription termination sites [34].

Other kinetoplastid parasites, *Tryp*. *cruzi* and *Leishmania major*, also encode homologous bromodomain containing proteins for BDF1-7, none of which have been characterized in *L*. *major* thus far. TcBDF2 contains an N-terminal bromodomain and binds acetylated histones at H4K10ac and H4K14ac and has also been shown to interact with histone H2B.V [35,36]. Expression of TcBDF2 accumulates following UV radiation in epimastigotes, suggesting that TcBDF2 could play a role in the DNA damage response [35].

For the majority of organisms discussed in this review, bromodomains make up the second largest group of epigenetic reader proteins, highlighting their importance in parasite biology (Fig 2). Interestingly, *Tri*. *vaginalis* has over 10 times as many bromodomains as any other protozoan parasite, making up over 50% of their readers. The expansion of not only their bromodomains but also their chromodomains suggests that the parasite has evolved divergent epigenetic regulatory pathways that rely heavily on recognition of PTMs. Alternatively, this expansion may be due to duplication events in the genome, generating pseudogenes that do not yield functional gene products. The latter hypothesis is unlikely, as the expansion is restricted to just bromodomain and chromodomain encoding genes, although further transcriptional and proteomic analysis will be necessary to determine this.

### Bromodomain proteins with functions outside the nucleus

Acetylation of lysine residues does not only occur in the nucleus. It is now clear from many proteomic analyses in different protozoan parasites that lysine acetylation is an abundant PTM that occurs on proteins involved in many cellular processes and in many cellular compartments [8,11,37–39]. Although most bromodomain proteins are nuclear and have been ascribed a nuclear function, there is some evidence of bromodomains with extranuclear function in protozoans.

Two nonnuclear bromodomain-containing proteins have been characterized in *Tryp*. *cruzi*, TcBDF1 and TcBDF3. TcBDF1 contains a C-terminal bromodomain and is localized to the glycosome, an organelle unique to trypanosomes that houses glycolytic enzymes [40]. TcBDF1 is present in all life cycle stages but is expressed at higher levels in the infectious trypomastigote stage. Overexpression of TcBDF1 resulted in replication defects leading to parasite death, suggesting that levels of TcBDF1 must be precisely maintained for optimal parasite fitness [40]. TcBDF3 is expressed in epimastigotes and localizes to the cytoplasm and flagellum, where it interacts with acetylated α-tubulin, an important structural modification of flagellar microtubules. An overexpressed mutant of truncated TcBDF3 that is missing the bromodomain is unable to bind acetylated lysines and resulted in aflagellar parasites with impaired growth in the epimastigote stage [41]. In *Tryp*. *brucei*, TbBDF4 localizes to both the nucleus and cytoplasm [34]. While in the nucleus TbBDF4 binds to regions of chromatin associated with transcriptional start sites, it is possible that it also has a regulatory role in the cytoplasm [34]. Alternatively, regulation of TgBDF4 trafficking between the cytoplasm and the nucleus could be a mechanism to control TbBDF4 function. The extracellular, gastrointestinal pathogen *E*. *histolytica* expresses a bromodomain protein, EhTAF1, which has been localized to both the nucleus and cytoplasm. EhTAF1 contains a C-terminal bromodomain and a domain of unknown function, consistent with the domain architecture found in other TAF1 proteins [42]. Immunoprecipitation experiments revealed that EhTAF1 interacts with EhTBP and EhTRF, both conserved components of the TFIID complex, suggesting that it is likely involved in transcriptional initiation in *E*. *histolytica*. Unlike other TAF1 proteins, EhTAF1 is significantly up-regulated upon heat shock stress conditions and is overexpressed in the cytoplasm and nuclei of trophozoites during these conditions. EhTAF1 partially colocalizes with EhHSP70 in the stress granules during heat shock stress [42], indicating that EhTAF1 has a nonconventional function in the heat shock stress response in addition to a conserved role in transcriptional regulation in *E*. *histolytica*.

### YEATS domains

Acetylation is by far the most abundant acylation that can occur on lysine, but it is not the only modification. Many different acylation marks have now been reported to modify histone lysines in eukaryotic organisms, including propionylation, butyrylation, succinylation, malonylation, glutarylation, and crotonylation [43]. A number of these modifications have also been identified in protozoan pathogens [39,43–47]. Bromodomains have low binding affinity for these larger acylation marks; however, the YEATS (Yaf9, ENL, AF9, Taf14, Sas5) domain is the first reader module identified to bind crotonylation [48]. Comprised of approximately 120 to 140 amino acids, the YEATS domain is highly conserved among eukaryotes. The domain contains an immunoglobulin fold containing a 2-layer beta-sandwich and 8 antiparallel beta strands (Fig 1). Several conserved amino acid residues form a flat shape on its binding groove, allowing recognition of different acyl-lysine residues [48]. YEATS domain-containing proteins have been identified as components of several complexes and therefore have been implicated in molecular functions including transcriptional regulation, DNA damage repair, and chromatin remodeling [48–50].

YEATS domains have been identified in protozoan genomes, although the number is reduced compared to bromodomains (Fig 2). Based on protein sequence, they have been designated as homologs of the YEATS protein family that includes human Gas41 and yeast Yaf9. In humans, Gas41 is a member of the Tip60 and SRCAP chromatin remodeling complexes and is responsible for deposition of H2A.Z onto chromatin [51]. In yeast, Yaf9 is a part of the NuA4 acetyltransferase and SWR1 chromatin remodeling complexes [52].

Two YEATS domain-containing proteins have been identified in *Tryp*. *brucei*, TbYEA1 (also named TbSWRC1 in [53]) and TbYEA2 that appear to be involved in chromatin remodeling. TbYEA1, revealed to be a Gas41/Yaf9 homolog by structural analysis [53], is associated with the methyl-lysine reader protein TbZCW1 and the bromodomain protein TbBDF2 [34]. Further co-immunoprecipitation (co-IP) experiments showed that TbYEA1 interacts with TbRuvB2, a helicase protein that is conserved in the SWR1 chromatin remodeling complex [53]. Depletion of TbYEA1 resulted in decreased chromatin-associated H2A.Z, indicating that TbYEA1 contributes to H2A.Z incorporation and may be a part of the SWR1 complex [53]. The second YEATS protein, designated as TbYEA2, is another potential Gas41/Yaf9 homolog that is associated with TbBDF6, TbEAF6, and TbHAT1 [34]. EAF6 homologs are found in the NuA4/Tip60 KAT complexes, suggesting that TbYEA2 is a member of a similar *Tryp*. *brucei* KAT complex. Additionally, Vellmer and colleagues found that TbYEA2 interacts with hypothetical protein Tb927.9.2910, a protein annotated as an NuA4 subunit [53]. This suggests that TbYEA1/SWRC1 and TbYEA2 likely contribute to SWR1 and NuA4/Tip60-like complexes in *Tryp*. *brucei*, respectively, and may have conserved functions as Gas41/Yaf9 homologs.

A single YEATS domain-containing protein is found in *P*. *falciparum*, PF3D7_0807000. Interactome analysis by co-IP showed that PF3D7_0807000 associates with PfRuvB1, PfRuvB2, and PfRuvB3 [54]. Homologs of these proteins are also members of NuA4/Tip60 KAT complexes in humans and yeast. This suggests that the function of YEATS domain-containing proteins is conserved in Apicomplexans. Although the specific reader characteristics of these proteins have yet to be characterized in protozoans, their conservation with homologs in other eukaryotes will help to elucidate a potential function of these YEATS domain-containing proteins.

### Methylation and epigenetics

The presence of methyl groups on DNA, mRNA, and histones is a well-recognized modification contributing to the regulation of gene expression and the structure and maintenance of chromatin. The importance of methylation and its regulators to cellular biology is evident by their conservation throughout eukaryotes.

An important distinction must be made between DNA methylation, mRNA methylation, and methylation of histones. The 3 modifications are regulated by independent factors but functionally cooperate in epigenetic regulation. Methylated DNA refers to the addition of a single methyl group on the fifth carbon (C5) of cytosine, forming 5-methylcytosine (5MC). DNA methyltransferases (DNMTs) add the methyl group, and a series of chemical reactions is required to revert the base back to unmodified cytosine. DNA methyl readers have a methyl-cytosine binding domain (MBD) and upon DNA binding initiate downstream signaling to regulate chromatin structure and gene expression. The presence of DNA methylation in many protozoans has been uncertain, only recent advances in technology have allowed for the sensitive detection of low levels of cytosine and cytosine-like methylation. Low levels of 5MC have now been detected in *Toxoplasma* [55], but little or no 5MC has been identified in *Leishmania* or trypanosomes [56,57]. While *Plasmodium* has almost no 5MC, Hammam and colleagues identified a more abundant 5hmC-like (5-Hydroxymethylcytosine) modification in the parasite’s genome [58]. Interestingly, many of these parasites possess conserved homologs of DNMTs, but no DNA methyl readers have been identified.

More recently, the significance of mRNA methylation has been revealed in protozoans [59–66]. mRNA adenosines, particularly in the 3′ UTR, can be methylated at the N6 position (m6A) to regulate mRNA structure and recruit effector proteins. Methylated mRNA is recognized and bound by the YTH (YT521-B homology) domain, which is present in *Plasmodium* species, *Toxoplasma*, and *Th*. *annulata* (S1 Table). Two YTH homologs have been identified in *P*. *falciparum* that are both expressed in the intra-erythrocytic stages [60,62]. Knockdown of PfYTH.2 revealed a role in translational repression that is essential for blood stage development [62]. Two YTH homologs have also been reported in *Toxoplasma* [59,61], one of which, TgYTH2, is also involved in repression of translation and is essential in the tachyzoite stage [59,61]. The divergence of these methyl-RNA readers from humans and their critical function in Apicomplexans hints at therapeutic potential for parasitic infections. In kinetoplastid parasites, mRNA methylation has been detected in both insect and mammalian stages of *Tryp*. *brucei* [65,66]. In the mammalian bloodstream forms of *Tryp*. *brucei*, the mRNAs coding for VSGs is heavily methylated, suggesting a regulatory role of this modification in antigenic variation [66]. The YTH domain is not conserved in kinetoplastids; however, other RNA binding domains may function as RNA methyl readers. Liu and colleagues identified *Tryp*. *brucei* TRRM2, which has an RNA binding domain, as a potential m6A reader [65].

Histone methylation and its regulators are essential and abundant in protozoan parasites [67–69]. In contrast to acetylation, addition of a methyl group does not alter the charge of an amino acid. The marks serve as binding sites for adaptor proteins that maintain or alter the chromatin environment and recruit additional factors. As a result, methyl reader proteins are critical mediators of signaling in response to histone methylation, and this is evident in the diversity and abundance of methyl reader domains. Methylation of histones is associated with both active transcription (H3K4me2, H3K4me3) and gene repression (H3K9me3, H3K36me2, H3K36me3), and many conserved methyl marks have been identified in protozoan parasites [47,68,70–76]. Remarkably, a few unique methyl marks have been identified whose functions remain unknown, hinting at the presence of divergent methyl reader proteins.

### Histone methyl readers

Methylated histones can be recognized and bound by a variety of protein domains including chromodomains, Tudor, PHD, PWWP, WD40, and some non-PHD zinc fingers (Table 1). The characteristic feature of these domains is an “aromatic cage” made up of 1 to 4 aromatic residues that creates a binding pocket around the target methyl group [77]. Differences between reader proteins determine the methylated residues they can bind (lysine, arginine) in specific methylated states (me1, me2, me3). Protozoan parasites possess all the aforementioned methyl-binding domains; however, PWWP domains are only found in some species. A large number of WD40, non-PHD zinc finger, ankyrin, and MBT containing proteins are present in protozoans, but these domains are capable of binding many different targets that include protein, DNA, and RNA. To date only one, the zinc-finger CW domain of ZCW1 in *Tryp*. *brucei* has been identified as a methyl lysine reader, interacting with a methyltransferase (SET26) and other chromatin factors at promoters and gene bodies [34]. This review will focus on the chromo, Tudor, PHD, and PWWP domains.

### Chromodomains

Chromodomains (chromatin-organization-modifier domains) are conserved in protozoan parasites and are the most well-characterized methyl reader. The domain, consisting of 30 to 70 amino acids forming a 3-stranded beta-sheet and an alpha-helix (Fig 1), can bind mono-, di-, or tri-methylated lysines. The function of chromodomains throughout eukaryotes is to regulate transcription, primarily through gene repression. Heterochromatin protein 1 (HP1) is a highly conserved chromodomain-containing protein that contains a single chromodomain at the N-terminus. Across many eukaryotic species, HP1 is consistently shown to bind H3K9me3 in heterochromatin and associate with other heterochromatin factors [78]. To initiate, propagate, and maintain heterochromatin, HP1 binds H3K9me3 and recruits methyltransferases that methylate nearby lysines, thus creating additional HP1 binding sites. *P*. *falciparum* encodes an HP1 homolog (PfHP1) that has been extensively studied and is critical for parasite replication and gene silencing [79]. Unlike most eukaryotes, where HP1 localizes to centromeres and telomeres, PfHP1 is found at subtelomeric regions and var genes during the intra-erythrocytic developmental cycle [80,81]. During asexual replication in the bloodstream, *P*. *falciparum* parasites express a single active var gene while the remaining var genes are repressed. PfHP1 binds the repressive H3K9me3 mark at inactive var genes and is critical for repression of these gene loci to maintain mutually exclusive var gene expression [79]. The role of PfHP1 during the switch from inactive to active var gene expression remains unknown.

PfHP1 is also involved in repression of the transcription factor PfAP2-G and consequently the prevention of sexual development, underscoring its important role in regulation of the *P*. *falciparum* life cycle [79]. Differentiation from asexual parasites to sexual gametocytes involves a restructuring of chromatin and also a relocalization of PfHP1 [82]. For example, in gametocytes, PfHP1 is found at a number of genes involved in host cell remodeling while it is absent from these genes in asexual parasites. While PfHP1 occupancy changes little during asexual stages, it is not surprising that major changes occur during sexual differentiation when a different subset of genes must be repressed by the presence of PfHP1 and genes involved in sexual differentiation, such as PfAP2-G, must be activated by PfHP1’s absence. *P*. *falciparum* possesses another protein with an N-terminal chromodomain (PF3D7_1140700), which also binds H3K9me3 [83]. It was recently found to be located in gene bodies of the *rifins*, *var*, and *stevor* virulence genes, and deletion of the chromodomain caused up-regulation of those genes, demonstrating the role of PF3D7_1140700 in repressing virulence factors [84].

In contrast to *Plasmodium* species, other protozoans, including related Apicomplexans, do not possess a conventional HP1 homolog. *Toxoplasma*, *Tryp*. *brucei*, *Cryptosporidium parvum*, and *Giardia* have chromodomains, but they are within proteins that contain different domain architectures than that of HP1. Interestingly, 2 proteins are encoded in the *Toxoplasma* genome (TGME49_268280 and TGME49_269760) that contain a single C-terminal, instead of the typical N-terminal chromodomain. Gissot and colleagues found that TGME49_268280 (TgChromo1) binds H3K9me3 and localizes to heterochromatin in centromeres and telomeres, similarly to HP1 homologs [85]. *Th*. *annulata*, *L*. *major*, and *Tryp*. *cruzi* each have a single protein with an N-terminal chromodomain, but their similarity to HP1 has not yet been explored (TA02685, LmjF.14.0160, TcCLB.506605.139). As observed in *P*. *falciparum*, *Entamoeba* species have 2 likely HP1 homologs, and *Tri*. *vaginalis*, consistent with its other epigenetic readers, has an unusually large number of predicted HP1 homologs.

Chromodomains are also found in lysine acetyltransferase MYST family members and the chromatin remodeling ISW1/SNF2 proteins. The MYST chromodomain is unique in that the structure includes an additional Tudor knot within the typical chromodomain beta sheet. How this modification affects binding and downstream function is unknown. As lysine acetyltransferases, the MYST family has primarily been studied for their role in histone acetylation, and the role of the chromodomain in this process is unclear. Determining the target binding site of the MYST chromodomain would reveal if it recruits the complex to an activating or repressive methyl mark. All protozoan parasites surveyed in this review encode at least 1 chromodomain-containing MYST, and *Tri*. *vaginalis* has an overabundance. ISW1/SNF2 chromodomain proteins are also found in most protozoans but are absent in the kinetoplastids. The precise function of ISW1/SNF2 proteins in protozoans has yet to be revealed but based on the conservation of these proteins with human and yeast homologs, it is likely that they are nucleosome remodelers that catalyze ATP-dependent alterations in nucleosomal DNA structure [86]. The absence of chromodomain-containing ISW1/SNF2 proteins in kinetoplastids suggests the presence of divergent nucleosome remodelers that do not contain familiar reader domains. Other putative methyl reader proteins that contain double chromodomains have been identified in protozoan parasites via domain searches but have not yet been characterized (Table 1). However, the high overall conservation and number of chromodomain-containing proteins in protozoan parasites hints at their critical nature and the breadth of their involvement in gene regulation and chromatin stability.

### Tudor domains

Tudor domains are relatively small reader domains that consist of approximately 60 residues forming a 5-stranded beta-barrel fold with 1 or 2 alpha-helices (Fig 1). Unlike other methyl readers discussed in this review, they can recognize both methylated arginine and methylated lysine. In general, methylated arginine is not common or nonexistent in many protozoan parasites [87,88]. For those that do possess methylated arginines, binding by specific Tudor domain-containing proteins has not yet been identified. Several protozoan parasites encode a Tudor staphylococcal nuclease (TSN) homolog (S1 Table). Interestingly, in *Toxoplasma* and *P*. *falciparum*, this homolog interacts with an arginine methyltransferase but binds RNA instead of methylated arginines [89,90]. These complexes are thought to be involved in RNA splicing, not epigenetic regulation. Similarly, the TSN homologs in *Tryp*. *brucei* and *E*. *histolytica* do not bind methylated histones but are reported to bind DNA [91,92]. Taken together, this evidence suggests that the TSN family is not specifically involved in epigenetic gene regulation in protozoan parasites. The “survival of motor neuron” (SMN) homologs also contain Tudor domains and have been identified in the Apicomplexans, but none of these have been characterized for their methyl-binding ability. *Tri*. *vaginalis* and *Giardia lamblia* both encode proteins that are annotated to contain a “SAGA associated factor 29” Tudor domain, the function of which is unknown. In humans and yeast, the Tudor-containing SAGA associated factor 29 is a component of the SAGA lysine acetyltransferase complex responsible for recruitment to H3K4me3 [93]. However, the *G*. *lamblia* and *Tri*. *vaginalis* homologs display minimal homology to the human and yeast proteins and may have an alternative function in these parasites. Tandem tudor domains (TTDs), which bind H3K9me3 in humans, do not appear to be conserved in the protozoans discussed in this review.

### PHDs

The PHD is a versatile domain found in hundreds of different proteins with a broad range of substrates, including unmethylated, methylated, and acetylated lysines. They are the most abundant reader, making up over 38% of all readers in each protozoan parasite, with the exception of *Tri*. *vaginalis* (Fig 2). The PHD is comprised of 50 to 80 residues with a zinc-binding motif (Fig 1). PHDs are commonly found in transcription regulatory complexes, and this is consistent with what has been observed in protozoan parasites [34,83,94]. Several parasite histone methyltransferases, demethylases, and SWI/SNFs contain a PHD (S1 Table). In *P*. *falciparum*, PHD1 and PHD2 appear to participate in separate GCN5/ADA2 complexes associated with H3K4me2 and H3K4me3 transcription activation marks [83]. Staneva and colleagues identified *Tryp*. *brucei* PHD1 in a complex similar to the piccolo-NuA4 complex in yeast [34]. The PHD of the *P*. *falciparum* methyltransferase PfSET10 mediates binding to non-methylated and mono-methylated histone H3K4 [95]. The PHD domain in PfSET2 binds H3K36me2 at var genes, thereby allosterically promoting PfSET2 methylation to create the repressive H3K36me3 mark and suppress gene expression [96]. Aside from this role in antigenic variation, the specific functions of PHDs in protozoan transcription regulatory complexes and their direct impact on parasite gene expression is unknown. Notably, in several eukaryotes, proteins with a DPF can bind both acetylated and methylated lysines [97]. While the conserved DPF domain has not been identified in protozoans, proteins with tandem PHD domains have been annotated whose functions have not been studied.

### PWWP domains

PWWP methyl reader domains are less abundant and poorly understood in eukaryotes. The domain is made up of a 5-stranded beta-sheet and a variable helical bundle with a characteristic Proline-Tryptophan-Tryptophan-Proline (PWWP) motif (Fig 1). Because these domains are capable of binding DNA in addition to methylated lysines, they are often found in DNA-binding transcription factors. This domain is even more scarce in protozoans; PWWP domains have only been identified in *Toxoplasma*, the kinetoplastids, and *E*. *histolytica* (Fig 2 and S1 Table). *Tryp*. *brucei* encodes 2 proteins that contain PWWP domains (TFIIS2-1 and TFIIS2-2). However, TFIIS2-1 was unable to bind DNA or methylated histones [98]. TFIIS2-2, on the other hand, can bind H4K17me3 and H3K32me3 but not DNA in vitro [99]. TFIIS2-2 was also found associated with RNAPII subunits and PAF1 at transcription terminations regions in *Tryp*. *brucei* [34]. The undefined role and paucity of PWWP domains provides little insight into the importance of these methyl readers in protozoan parasites.

### Histone phosphorylation readers

In addition to being acetylated and methylated, histones can also be phosphorylated on serines, threonines, and tyrosines. Histone phosphorylation is less abundant and primarily studied for its participation in the DNA damage response pathway; however, it also plays a role in gene regulation in eukaryotes [1]. Although a dedicated histone phosphorylation reader module has not yet been identified, there are a few examples of proteins that are recruited to phosphorylated histones [100]. The 14-3-3 protein family, which is abundant in all eukaryotes, including protozoans, is primarily cytoplasmic, mediating protein–protein interactions in signaling cascades [101]. Although 14-3-3 proteins can have many different binding targets including both modified and unmodified proteins, they can also function as PTM readers [101,102]. The *P*. *falciparum* 14-3-3 protein (Pf14-3-3I) localizes to both the nucleus and cytoplasm and is recruited to parasite histones by binding H3S28ph [103]. The protein had low affinity for another phosphorylated serine on histone H3, indicating that this is a specific interaction with phosphorylated serine 28. The regulatory significance and the downstream implication of Pf14-3-3I association with chromatin remains unknown. While the 14-3-3 domain is conserved in all protozoans discussed in this review, to date, it has only been examined in *P*. *falciparum*. Two other common eukaryotic histone phosphorylation reader domains are the BIR and BRCT domains. Although the conserved BRCT domain has been identified in protozoans, their phospho-histone binding capabilities are unknown, and the parasites possess no identifiable BIR domains.

### Epigenetic readers as drug targets

Epigenetic factors have been intensively studied due to their relevance to human disease. The enzymatic writers and erasers have historically been the focus of therapeutic intervention; however, epigenetic readers have also been under scrutiny for their potential as drug targets. Small molecule inhibitors of reader proteins have shown promise as treatments for human cancers, and many are currently in clinical trials [104]. Bromodomain inhibitors have shown the most promise, likely due to the structure of their conserved binding pocket and the ability of inhibitors to bind with high affinity. Other readers, including YEATS domains and methyl-binding domains, present more of a challenge to drug development as they are generally more promiscuous and often bind multiple targets with varying levels of affinity. However, some success has been achieved with high-throughput screening and fragment-based NMR screening to identify high-affinity and selective inhibitors of methyl readers [105]. The human epigenetic reader inhibitors currently in development are reviewed in Arrowsmith, Engelberg, Teske, and Zhu [77,104–106].

Reflecting efforts to target human reader proteins, bromodomains are proving to be amenable therapeutic targets for parasites. A screen of epigenetic inhibitors against *P*. *falciparum* identified 6 and 3 reader protein inhibitors that were active against asexual and sexual stages, respectively [107]. Another screen of bromodomain inhibitors against *P*. *falciparum* blood stages identified a number of compounds with some potency against the parasite, including the inhibitor SGC-CBP30, which had 7-fold selectively for the parasite over human cells [108]. The bromodomain inhibitor I-BET151 that targets the BET family of bromodomain proteins in humans inhibits *Toxoplasma* tachyzoite growth at concentrations lower than levels that are toxic to human cells [11]. This compound also targeted bromodomain proteins in *Tryp*. *cruzi* and *Tryp*. *brucei*, to inhibit parasite growth and development [32,109]. The acetylinodolizine compound GSK2801 was also shown to inhibit *Tryp*. *brucei* growth, by binding TbBDF2 [30]. Alonso and colleagues developed a fluorescence-based in vitro assay to test inhibitor binding to TcBDF3 [109]. Binding of small molecules to the bromodomain quenches the intrinsic fluorescent signal from the aromatic residues within the binding pocket. Using this approach, they identified compounds I-BET151 and JQ1(+) as inhibitors of TcBDF3. Another bromodomain inhibitor, L-Moses, which was developed to target the bromodomain in the human lysine acetyltransferase GCN5, also binds the bromodomain of GCN5 homologs in *Toxoplasma* and *P*. *falciparum*, decreasing parasite viability [24,110].

The YEATS domain has recently been targeted for small molecule drug design, and inhibitors to human AF9 and ENL, YEATS-containing proteins, have shown significant promise [110,111]. YEATS inhibitors have yet to be tested against protozoans; however, the current AF9/ENL inhibitors may not have high specificity for the only YEATS homologs (Gas41 and Yaf9) identified in these parasites. Inhibitors to other histone reader proteins, including methylation and phosphorylation readers, have not been investigated for their antiparasitic potential.

Although bromodomains have been the first reader modules that have been successfully targeted with small molecule inhibitors, understanding the biology of other reader proteins and their critical functions in epigenetic pathways will undoubtedly reveal novel approaches for antiparasitic therapies.

## Conclusions

There are many examples of how protozoan pathogens rely on epigenetic mechanisms to infect and cause disease in their hosts. The study of transcriptional mechanisms in these divergent organisms continues to provide insight into how they regulate critical pathways throughout their life cycle and reveal novel ways of fighting parasitic infections. Until recently, the PTM reader proteins have been an unexplored trove of opportunity for understanding these regulatory mechanisms. Analyzing epigenetic readers has led to discoveries of many other critical transcription-related factors, and the intricate network between DNA-binding proteins, chromatin-modifying complexes, and transcription machinery is being unveiled. Bromodomains, in particular, have shown significant promise for drug development, and the lessons learned are being applied to other reader proteins. As we learn more about the targets, binding partners, and functional contributions of individual reader proteins, we build a profile of the capabilities of the many different epigenetic regulatory complexes in protozoans. In the case of proteins, or complexes with multiple reader domains, this will also support our understanding of the dynamics of complex recruitment to histones with multiple PTMs. As the implications of individual modifications are understood, we can then determine how the presence or absence of a particular modification influences the deposition, or removal of another modification (PTM crosstalk). Furthermore, competition for the same target residues (for example, acetylation versus methylation of lysines) must also be considered as another level of regulation of gene expression. Ongoing efforts will reveal the similarities and divergence between protozoan parasites and their hosts, allowing for multiple avenues for development of therapeutics.

## Supporting information

S1 Table Known reader proteins of protozoan parasites. (XLSX)
